# The Efficacy of Body-Weight Supported Treadmill Training and Neurotrophin-Releasing Scaffold in Minimizing Bone Loss Following Spinal Cord Injury

**DOI:** 10.3390/bioengineering11080819

**Published:** 2024-08-12

**Authors:** Michael Weiser, Lindsay Stoy, Valerie Lallo, Sriram Balasubramanian, Anita Singh

**Affiliations:** 1 School of Biomedical Engineering, Science and Health Systems, Drexel University, Philadelphia, PA 19104, USA; 2 Biomedical Engineering, Widener University, Chester, PA 19103, USA; 3 Bioengineering, Temple University, Philadelphia, PA 19122, USA

**Keywords:** spinal cord injury, neurotrophins, spine rehabilitation, biomechanics, bone

## Abstract

Spinal cord injury (SCI) can lead to significant bone loss below the level of the lesion increasing the risk of fracture and increased morbidity. Body-weight-supported treadmill training (BWSTT) and transplantation strategies using neurotrophins have been shown to improve motor function after SCI. While rehabilitation training including BWSTT has also been effective in reducing bone loss post-SCI, the effects of transplantation therapies in bone restoration are not fully understood. Furthermore, the effects of a combinational treatment strategy on bone post-SCI also remain unknown. The aim of this study was to determine the effect of a combination therapy including transplantation of scaffold-releasing neurotrophins and BWSTT on the forelimb and hindlimb bones of a T9-T10 contused SCI animals. Humerus and tibia bones were harvested for Micro-CT scanning and a three-point bending test from four animal groups, namely injury, BWSTT (injury with BWSTT), scaffold (injury with scaffold-releasing neurotrophins), and combinational (injury treated with scaffold-releasing neurotrophins and BWSTT). BWSTT and combinational groups reported higher biomechanical properties in the tibial bone (below injury level) and lower biomechanical properties in the humerus bone (above injury level) when compared to the injury and scaffold groups. Studied structural parameters, including the cortical thickness and bone volume/tissue volume (BV/TV) were also higher in the tibia and lower in the humerus bones of BWSTT and combinational groups when compared to the injury and scaffold groups. While no significant differences were observed, this study is the first to report the effects of a combinational treatment strategy on bone loss in contused SCI animals and can help guide future interventions.

## 1. Introduction

Spinal cord injury (SCI) is a common cause of morbidity with a prevalence of 302,000 cases with an incidence of 18,000 new cases annually in the United States [1]. In addition to sensory and motor deficits, SCI also leads to bone loss below the level of lesion, resulting in an increased risk of fracture and greater morbidity [2]. The degree of bone loss is greater in complete SCI as compared to incomplete SCI, with the latter being more common [1,2]. Greater bone loss in complete SCI could be attributed to the increased immobility-related loss of mechanical stimuli, which has a strong influence on bone integrity and structure. Reported changes in the skeletal structure include changes in the cortical and trabecular regions of the bones following SCI [3]. Furthermore, the reported loss of bone mineral density (BMD) has been reported to stem primarily from the degradation of trabecular bone [4,5].

Current treatment for SCI includes rehabilitation therapy such as body-weight-supported treadmill training (BWSTT). Through BWSTT, synapses and motor tasks can be recovered below an incomplete SCI [6]. However, recovery of motor function following a complete SCI has not been demonstrated [7]. Furthermore, expected motor recovery following incomplete SCI is directly related to the number of spared descending pathways [7]. Several clinical and animal studies support the role of BWSTT in restoring motor function while reducing muscle atrophy and bone loss with improvement in BMD [8,9,10]. Giangregorio et al. (2005) studied five human participants with SCI who underwent BWSTT over 6–8 months and noted a range of increased muscle cross-sectional areas from 3.8% to 56.9%. While BMD was reduced in all participants’ lower limbs ranging from −1.2% to −26.7%, the participant displaying the greatest ambulatory function demonstrated the smallest reduction in BMD, and the participant who completed the fewest BWSTT sessions demonstrated the greatest reduction in BMD [8]. In another study, Shields et al. (2006) studied the effects of unilateral electrical stimulation of soleus muscles, simulating training response, in patients with SCI. Stimulation delivering a compressive force of 1.5 times the body weight on the tibia for three years resulted in a 31% increase in trabecular BMD at the distal tibia of trained limbs compared to untrained limbs [11]. Thus, the role of BWSTT in improving bone morphology and strength after SCI along with motor and muscle functions is evident.

In addition to rehabilitation therapy, transplantation strategies is another investigational treatment modality that aims to promote neuroprotection and regeneration post-SCI. Bioengineering transplantation strategies including biomaterials such as polyethylene glycol (PEG) loaded with neurotrophins including brain-derived neurotropic factor (BDNF) and neurotrophin-3 (NT3) have reported promising outcomes [12,13,14]. After the initial insult to the spinal cord, a cascade of secondary events leads to an acute inflammatory response that induces further tissue damage. Targeting these secondary events to prevent additional tissue damage represents a promising strategy to improve patient outcomes. BDNF and NT3 are known to play a key role in minimizing secondary injury while promoting regeneration and sprouting of injured axons [6,15]. Piantino et al. (2006) studied axonal rewiring in rats that underwent spinal cord hemisection and implantation with NT3 delivered via hydrogels. They noted significant increases in motor function studied using BBB scores for NT3-treated animals (16.43 ± 0.86) compared to controls (13.75 ± 0.72) supported by significantly more axonal sprouting (*p* < 0.01) in the NT3 animals [15]. Another study demonstrated increased recovery of fine motor control in a cervical dorsolateral funiculotomy animal model when transplanted with neurotrophins loaded PNIPAAM-g-PEG versus PNIPAAM-g-PEG alone [16]. These available studies support the role of neurotrophins-loaded scaffolds in promoting motor recovery and regeneration of fibers post-SCI. However, no study has related the effects of these transplantation approaches on bone loss post-SCI. Since neuroprotection and regeneration post-transplantation of neurotrophins results in improved motor function, it can be hypothesized that the improved motor function will reduce bone atrophy. It remains unknown if the motor function improvements post-transplantation therapy are significant enough to reduce bone loss following SCI. Furthermore, the promising outcomes of BWSTT, the current standard of care for SCI subjects, and transplantation strategies holding the most promise in treating SCI warrant additional studies that investigate the effects of combinational treatment strategies combining these two strategies in treating SCI-related bone loss. The current study fills this critical gap and aims to determine the effects of a combinational therapy using transplantation of biomaterials loaded with neurotrophins at the SCI site in conjunction with BWSTT on structural and biomechanical properties of the forelimb (unaffected) and the hindlimb (affected) bones in a thoracic contusion SCI animal model. We hypothesize that the combination of these therapies will reduce bone loss below the level of the lesion (affected hind limb) when compared to no treatment and alone treatments. Furthermore, the combinational intervention will also result in reduced over-compensation of the unaffected limb when compared to no treatment and alone treatments.

## 2. Methods

Thirty-two adult female Sprague Dawley rats (body weight: 200–250 g) were used in the current study. Female rats were used due to our previous experience in handling them while performing treadmill training experiments. Animals were assigned to one of the four groups (n = 8 per group, power of 0.8, alpha and effect size of 0.05) listed in Table 1. The power analysis was performed based on a previously reported study by Tom et al. [7]. Group 1 (injury) was an injury group that received a T9/10 contusion SCI and no treatment. Group 2 (BWSTT) was the body-weight-supported treadmill training alone group that received contusion SCI and BWSTT as treatment. Group 3 (scaffold) was the transplantation alone group that received contusion SCI and an injection of poly-ethylene glycol (PNIPAAM-g-PEG) loaded with BDNF+NT3 neurotrophins as treatment. Finally, Group 4 (combinational) was the combinatorial group that received a contusion SCI followed by both transplantation (PNIPAAM-g-PEG releasing BDNF+NT3) followed by BWSTT as treatment. Group 2 and Group 3 received their therapies (BWSTT or transplant) one week post-contusion injury, while Group 4 received the transplant surgery one week post-contusion injury and then began BWSTT one week post-transplant surgery. Eight weeks after the surgery or last intervention in each group, the humerus and tibia bones were harvested from the fore and hind limbs of all animals, respectively (Table 2). The use of laboratory animals and all procedures were in accordance with the National Institute of Health and approved by the Institutional Animal Use and Care Committee. All efforts were made to minimize animal suffering during all procedures. Steps taken for animal care and to minimize pain throughout the study are included in the relevant sections below.

### 2.1. Spinal Cord Contusion Injury and Animal Care

A moderate contusion SCI at the T9/10 level was induced in all animals per the previously reported studies that have established BWSTT protocols for an animal in a bipedal position. The animals were anesthetized using a ketamine (76 mg/kg, Fort Dodge Animal Health, Fort Dodge, IA, USA), xylazine (7.6 mg/kg, Ben Venue 49 Laboratories, Bedford, OH, USA), and acepromazine maleate (0.6 mg/kg, Boehringer Ingelheim Vetmedica, Inc., St. Joseph, MO, USA) mixture by intraperitoneal injection. Once deeply sedated, the animals were then prepared for a T9-T11 laminectomy. The dura mater was exposed from the laminectomy procedure, an NYU impact device was used to induce a moderate spinal cord contusion injury by dropping a 10 g, 2 mm diameter rod from 25 mm above the dura mater. Subcutaneous fat was then placed on top of the injury site to prevent adherence. The back muscles were sutured and secured by wound clips, and the animals were then set on heating pads and observed until they regained consciousness. The rats’ water bottles were substituted with H_2_O hydrogels and long-stemmed water bottles, and their food was placed directly into the cage to accommodate for their decreased mobility. For the two weeks after injury, the animals were injected subcutaneously with 3–5 mL of saline twice a day, and ampicillin (0.1 cc, 22.7 mg/mL) once a day. Two to three weeks post-surgery, the rats’ bladders were manually expressed three times a day, and urine assessments were performed using urine strips to ensure the animals were recovering well and remained healthy. Animals that presented signs of abnormalities or infection were treated or removed from the study per the approved protocol.

### 2.2. Biomaterial Scaffold Transplantation Surgery

One week post-contusion SCI, animals in the scaffold group (Gr 3) and combinational group (Gr 4) received a second surgery for the transplantation of poly (N-isopropylacrylamide) grafted with polyethylene glycol (8000 g/mol) (PNIPAAM-PEG) loaded with neurotrophins (BDNF+NT3). Combinational treatment of BDNF+NT3 was chosen based on previously reported studies that have shown motor improvements post-this transplantation approach [12,13,14]. For this surgery, the animals (in Gr 3 and Gr 4) underwent another T9-T11 laminectomy to expose the dura mater around the original injury. The site of the injury was identified and an injection of a 5 μL solution of PNIPAAM-g-PEG loaded with co-dissolved BDNF (0.5 × 10^6^/5 μL) and NT3 (0.5 × 10^6^/5 μL) was injected by using a positive displacement pipette. Because of the thermosensitive properties of poly (N-isopropylacrylamide), the solution was kept on ice to ensure it maintained its liquid state; once injected, the solution became an elastic gel. The animals’ surgical sites were then closed using the same methods as the initial surgery and the animals received the same aftercare.

### 2.3. Behavioral Analysis

All animals were tested in an open field to measure their hindlimb function using the Basso, Beattie and Bresnahan (BBB) scale. Animals were allowed to move freely for 4 min each in an enclosure and then scored based on their hindlimb joint movements (hip, knee and ankle) from 0 (no movement of any joints) to 21 (normal movement of all three joints). The BBB tests were conducted before the injury (baseline), 2–3 days after injury, and then each week thereafter until the last time-point by two trained and blinded observers.

### 2.4. Body Weight Supported Treadmill Training (BWSTT)

One week after the initial contusion injury and one week after the transplantation, animals in Group 2 and Group 4 began BWSTT, respectively. These animals were placed in a vest that was attached to a body-weight support arm by Velcro. The arm was positioned over a treadmill and supported 75% of the animal’s body weight. Each animal in these groups walked 1000 steps per day at a speed of 7 cm/s, five days per week for eight weeks [6].

### 2.5. Bone Harvest

Eight weeks after injury (Gr 1) or the last intervention (Groups 2–4), all the animals were euthanized per the approved protocol and the humerus and tibia bones were harvested from the fore and hind limbs, respectively. For Group 1, the bones were harvested at Week 8. For the BWSTT (Gr 2) and scaffold (Gr 3) groups, the bones were harvested at Week 9 because these animals had the intervention (BWSTT or transplant) starting one week after the initial injury. For the combinational (Gr 4) group, the bones were harvested at Week 10 because these animals had two interventions, the last one (BWSTT) beginning two weeks post-initial contusion injury. During euthanasia, the animals were injected with 1 mL of Euthasol (Virbac AH, Fort Worth, TX, USA). The humerus and tibia bones were then removed from the fore and hind limbs, respectively. Harvested bones were stored in a freezer at −20 degrees Celsius until further scanning and biomechanical testing were performed.

### 2.6. Micro-CT Scanning

Before scanning, the bones were wrapped in parafilm and placed in a low-density plastic tube filled with PBS solution. The lid was secured with parafilm, and a specialized nut was used to secure the tube into the Micro-CT machine (Skyscan 1172 Micro-CT, Bruker Corporation, Billerica, MA, USA). The entire length of the humerus bone was scanned at a voltage and current value of 80 kV and 124 μA, respectively, with a resolution of 4000 × 2664 μm^2^ and a magnification of 3.48 μm. The entire length of the tibia bone was scanned at a voltage and current value of 65 kV and 156μA, respectively, with a resolution of 2000 × 1000 μm^2^ and a magnification of 4.9 μm. An aluminum 0.5 mm filter was used for both the humerus and tibia scans.

### 2.7. Micro-CT Analysis

**Figure 1:**
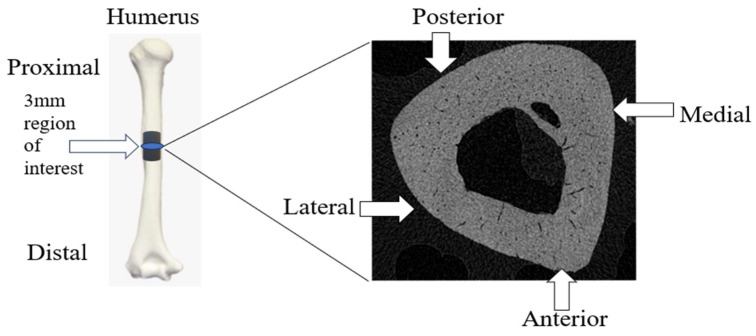
Region of interest (ROI) of humeral diaphysis and representative cross-sectional CT image.

**Figure 2:**
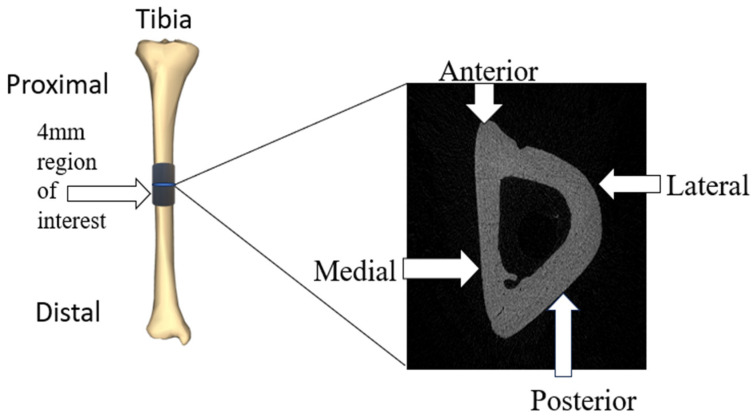
Region of interest (ROI) of tibial diaphysis and representative cross-sectional CT image.

After scanning, the images were reconstructed using NRecon Software (Microphotonics Inc., Allentown, PA, USA) [17]. Bone scan quality was improved by removing defects such as ring artifacts and beam hardening. After reconstruction, the diaphyseal regions were defined to draw the regions of interest (ROIs) in each area. ROIs were drawn on each image in the designated region to create volumes of interest (VOIs) using CTan Software (Bruker Corporation, Billerica, MA, USA). The humeral diaphyseal region was characterized as 1.5 mm above and below the midpoint of the bone (3 mm in total) (Figure 1). The tibial diaphyseal region was 2 mm above and below the midpoint of the bone (4 mm in total) (Figure 2). Once the VOIs were created, CTan Software (Bruker Corporation, Billerica, MA, USA) was utilized to run 2D and 3D binary analysis on the images to quantify the bone parameters including bone volume to total volume ratio (BV/TV) and the cortical thickness.

### 2.8. Three-Point Bending Biomechanical Testing and Analysis

**Figure 3:**
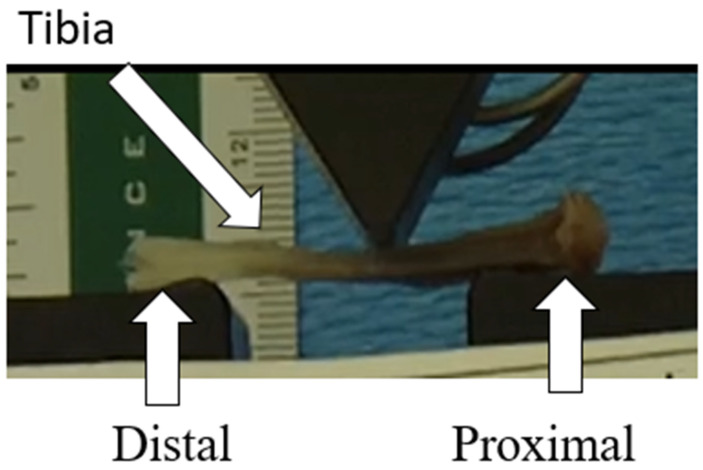
Three-point bending setup.

**Figure 4:**
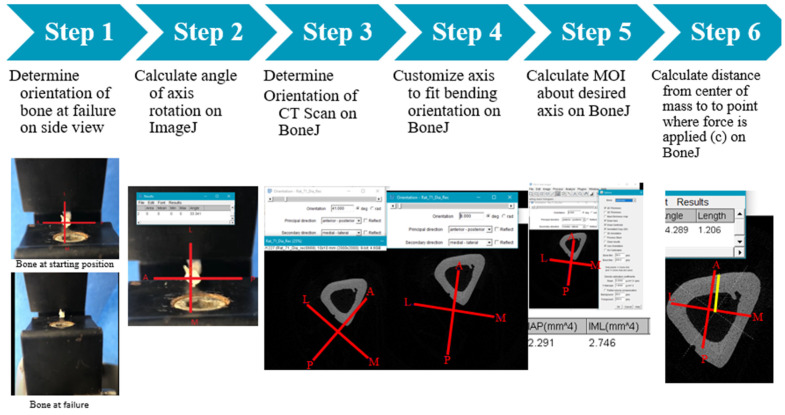
Detailed steps utilized to calculate MOI for each tested bone sample. Red lines are the orientation axis. Yellow line is the distance from the center of mass to th the point where force is applied as mentioned in Step 6.

After Micro-CT scanning, the bones underwent three-point bending biomechanical testing (Figure 3). Bones were tested until failure at a rate of 5 mm/min while the load–displacement data were acquired. To account for any rotation of bones during the test, videos (front and side/cross-sectional view) were captured to determine the specific bending axis when the bones failed [18,19]. These axes were used to calculate the bone-specific Moment of Inertia (MOI), which measures the capacity of a cross-section to resist bending, using the Micro-CT images and BoneJ Software (V7.0.19) [20] (Figure 4). The stress–strain parameters were then calculated using a customized MATLAB code (R2023a, MathWorks, Natick, MA, USA), and the obtained plots were used to determine each bone’s ultimate stress, strain at ultimate stress, ultimate load, and energy to maximum force, which was measured as the area under the load–displacement curve at maximum force [21,22].

### 2.9. Statistical Analysis

Statistical analysis was performed using SPSS (Version 11.5, IBM, Chicago, IL, USA), and significance was determined using a *p*-value of <0.05. The dataset was analyzed for distribution and the obtained normally distributed data was analyzed using two-way ANOVA for each studied parameter. A post hoc Bonferroni test was performed for multiple comparisons between groups. All values are expressed as mean ± standard deviation.

## 3. Results

### 3.1. Behavioral Test: BBB

Scores obtained from the two scorers were averaged. No significant differences in BBB scores were observed in any study groups post-injury.

### 3.2. Structural Parameters Obtained from Micro-CT Images

#### Cortical Thickness

**Figure 5:**
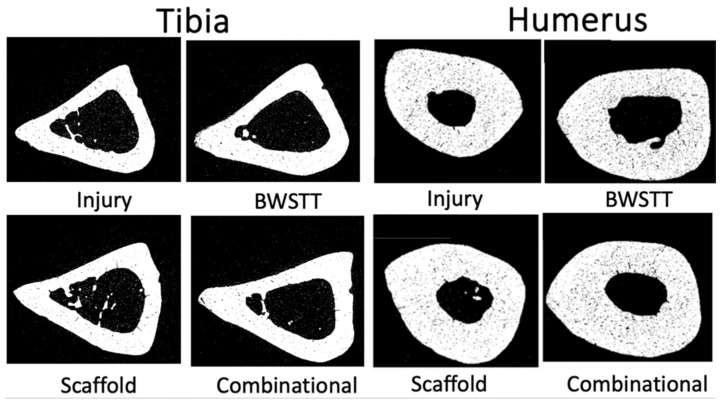
Exemplar Micro-CT images of the diaphyseal region of the tibia and humerus bones from each group.

**Figure 6:**
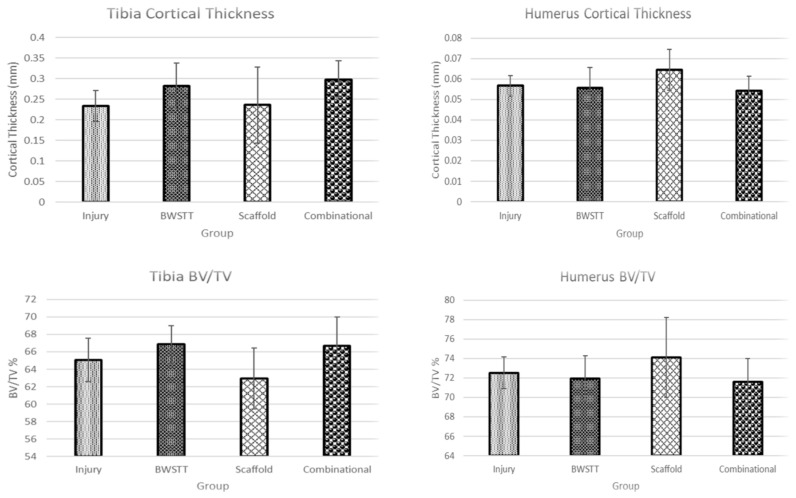
Structural parameters (cortical thickness and BV/TV) of tibia and humerus bones obtained from Micro-CT images. All values are expressed as mean ± SD. No significant differences were observed in any parameters between groups for both tibia and humerus bones (*p* > 0.05).

For the tibia, both the BWSTT (Gr 2) and combinational (Gr 4) groups had a greater diaphyseal cortical thickness than the injury (Gr 1) and scaffold (Gr 3) groups. The combinational group had the greatest overall cortical thickness, which aligns with our hypothesis that the extent of bone loss was expected to be minimal in the combinational group when compared to the no-treatment or alone-treatment groups (Figure 5 and Figure 6). For humeri, the scaffold group had the greatest cortical thickness. Both the combinational and BWSTT groups demonstrated less cortical thickness than the injury and scaffold groups, supporting our hypothesis of increased forelimb compensation in the injured and scaffold-alone SCI animals (Figure 5 and Figure 6). However, no observed differences were statistically significant.

### 3.3. Bone Volume to Total Volume Ratio (BV/TV)

For the tibia, the group with the greatest BV/TV was the BWSTT (Gr 2) followed by the combinational (Gr 4) group. Both groups demonstrated greater BV/TV when compared to the injury (Gr 1) and scaffold (Gr 3) groups. The observed BV/TV was lowest in the scaffold group (Figure 6). For humeri, the scaffold group had the highest BV/TV followed by the injury group. The lowest BV/TV was reported in the combinational group, indicating the lowest degree of forelimb compensation (Figure 6). However, no observed differences were statistically significant.

### 3.4. Biomechanical Parameters Obtained from Three-Point Bending Test

#### Moment of Inertia (MOI)

**Figure 7:**
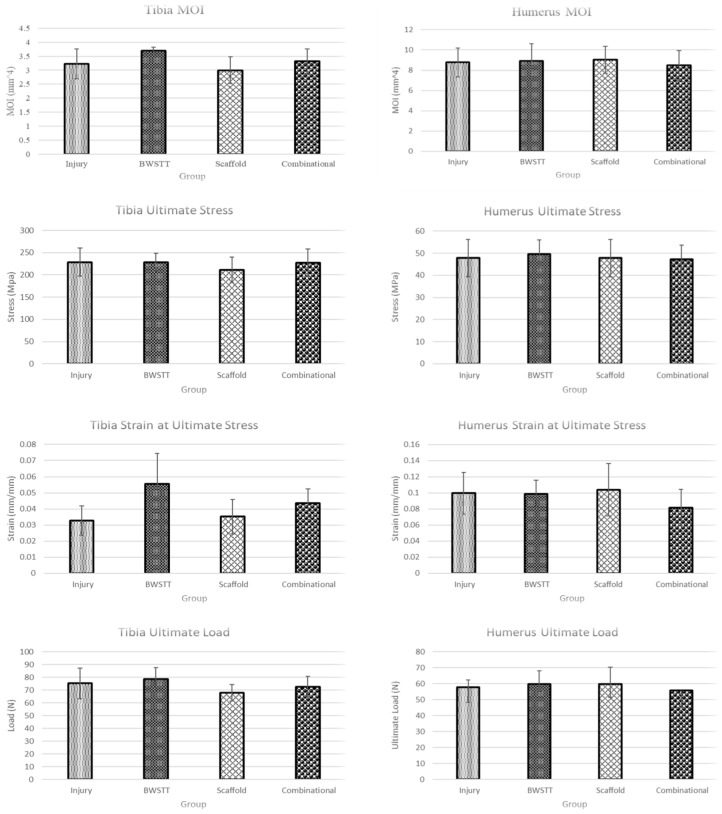

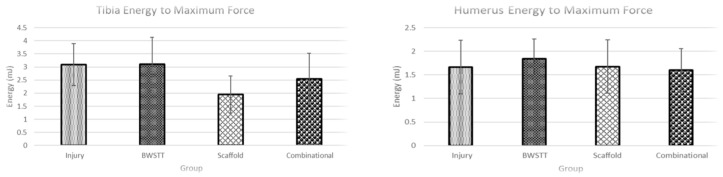
Biomechanical parameters (MOI, ultimate stress, strain at ultimate stress, ultimate load, and energy to maximum force) of tibia and humerus bones when subjected to three-point bending. All values are expressed as mean ± SD. No significant differences were observed in any parameters between groups for both tibia and humerus bones (*p* > 0.05).

For tibia, the group with the greatest MOI was the BWSTT (Gr 2) followed by the combinational (Gr 4) group. The scaffold (Gr 3) group and injury (Gr 1) group both were less than the BWSTT and combinational groups (Figure 7). For humeri, very little difference was noted between the four groups with the combinational group having the lowest MOI. This further supports the hypothesis that the combinational group had the lowest degree of forelimb compensation (Figure 7). However, no observed differences were statistically significant.

### 3.5. Ultimate Stress

For the tibia, there was very minimal difference in the ultimate stress values between groups except for the scaffold (Gr 3) group, which was the lowest (Figure 7). For humeri, average ultimate stress values were also minimally different from each other (Figure 7). No observed differences were statistically significant.

### 3.6. Strain at Ultimate Stress

For the tibia, the BWSTT (Gr 2) group had the greatest average ultimate strain, followed by the combinational (Gr 4) group. The injury (Gr 1) group demonstrated the lowest average ultimate strain (Figure 7). For humeri, the combinational group had the lowest average strain at ultimate stress. This suggests less forelimb compensation within this group. The remaining three groups had a minimal difference in ultimate strain (Figure 7). No observed differences were statistically significant.

### 3.7. Ultimate Load

For the tibia, the BWSTT (Gr 2) group displayed the greatest average ultimate load while the scaffold (Gr 3) group reported the lowest ultimate load (Figure 7). For humeri, the combinational (Gr 4) group reported the lowest average ultimate load. This supports our hypothesis of less forelimb compensation occurring in this group. The remaining three groups were minimally different from each other (Figure 7). No observed differences were statistically significant.

### 3.8. Energy to Maximum Force

For the tibia, the BWSTT (Gr 2) group was noted to have the greatest energy while the scaffold (Gr 3) group had the lowest energy (Figure 7). For humeri, there was a slight difference in groups with the combinational (Gr 4) group demonstrating the lowest average energy to maximum force (Figure 7). This suggests the combinational group underwent less forelimb compensation. However, no observed differences were statistically significant.

## 4. Discussion

The aims of this study were to evaluate the structural and biomechanical changes in the hindlimb (tibia) and forelimb (humerus) bones of thoracic contused SCI rats after various treatment approaches, namely BWSTT, transplantation using bioengineering scaffold-releasing neurotrophins, and combinational (including both transplant and BWSTT). Micro-CT imaging of the analyzed bones reported increased cortical thickness and BV/TV in the affected hindlimb bone (tibia), which was below the level of injury, of the combinational and BWSTT groups. Similar findings of active treadmill training improving bone quality have been reported previously in SCI animals. Yarrow et al. (2012) reported that quadrupedal BWSTT slowed the reduction in cortical bone area measured on Micro-CT following SCI [23]. In the current study, the BWSTT and combinational therapies were also effective in reducing forelimb (unaffected limb) overcompensation as evidenced by the lower cortical thickness and BV/TV in the humerus bones of the animals in these groups when compared to animals in the injury and scaffold alone groups. The findings from this study also reported that the transplant of scaffold-releasing neurotrophins alone was not effective in reducing bone loss in the hindlimbs or decreasing overcompensation-induced bone changes in the forelimbs. Previously reported transplantation studies have demonstrated PEG hydrogel loaded with BDNF to be effective in axonal regeneration. Grous et al. (2013) studied the use of PEG and BDNF on rats that underwent SCI and reported improved fine motor skills in addition to regenerating axons [24]. Tom et al. (2018) also reported the beneficial effects of a bioengineered scaffold loaded with neurotrophins and BWSTT in restoring H-reflex responses after SCI. While these studies report the efficacy of transplantation therapy on axonal regeneration, spasticity, and functional performance, they did not investigate the effects of transplantation therapy on bone changes [7]. Findings from the current study are the first to report the effects of transplantation therapy alone and in combination with BWSTT on SCI-induced changes in both the forelimb (unaffected) and hindlimb (affected) bones.

For the studied biomechanical parameters, our results supported the radiographic findings. Correlations between biomechanical and radiographic findings have been reported previously. Voor et al. (2012) reported a 67% loss in the BV/TV and a 50% reduction in the strength of the cancellous bone of contused SCI animals [24]. The beneficial effects of exercise including BWSTT, and other training have been reported to improve bone quality post-SCI. Zamarioli et al. (2013) studied the efficacy of standing frame therapy on maintaining bone biomechanics in rats that underwent SCI. They found that standing frame therapy following SCI attenuated the loss of stiffness in the femur and tibial bones of rats that underwent SCI without therapy [25]. Several other studies have confirmed the beneficial effects of training post-rehabilitation training in SCI subjects [26,27]. The current study confirms these findings. We found that the BWSTT and combinational groups had stronger tibia (affected hindlimb), as evidenced by higher MOI, ultimate stress, strain at ultimate stress, ultimate load, and maximum energy. These therapies also correlated with reduced forelimb overcompensation as the humeri in these groups were noted to exhibit weaker mechanical properties when compared to injury and scaffold alone groups. Also, the scaffold-alone group was not effective in strengthening the tibia, which aligns with our previously discussed results of lower cortical thickness and BV/TV.

Findings from our study confirm the previously reported beneficial effects of BWSTT in bone restoration. This study further provides evidence of combinational therapy including BWSTT and hydrogel-releasing neurotrophins to have a similar beneficial effect and offers promise to serve as a therapy that can minimize bone loss following SCI. Although the combinational group received an additional transplantation therapy of scaffold loaded with neurotrophins, it failed to have any additional benefit when compared to BWSTT alone treatment. We attribute this to the delayed BWSTT in the combinational group. Delayed rehabilitation training has been reported to have a determinantal effect on bone recovery as reported previously [23]. In the combinational group animals, a transplantation therapy was performed seven days post-contusion injury as supported by previously published work that reported the beneficial outcomes of delayed transplant in SCI animals [24,25,26,27,28,29,30]. However, this delayed transplantation imposed a longer immobilization period for the combinational group and further delayed (two-week delay) the onset of the BWSTT in this group. The two-week delay in starting the BWSTT in the combinational group animals could have resulted in no further improvement in bone restoration in this group. However, it is noteworthy that despite the delayed training, animals in the combinational group did report improvement in the affected bone quality and reduced overcompensation-based changes in the unaffected forelimb bone. This warrants future studies that include additional injury and sham groups that undergo BWSTT two weeks later thereby mimicking the transplantation surgery in the combinational group and allowing a time-matched comparison of the studied interventions.

The rationale for the current study was to fill the current gap in understanding the effects of combinational treatment strategies in treating SCI-related bone loss. This study offers an understanding of the effects of no, alone and combinational treatment approaches on bone loss in affected and unaffected limb post-moderate SCI. While the findings of this study are novel, there are some major limitations of the current study is that there was no age-matched control group, and only female animals were utilized in this study. Since no sex-related differences have been reported in bone loss studies reported previously [31], we consider the use of female rats that have been extensively used in the published BWSTT and transplantation studies and allowed the discussion of findings from the alone treatment groups of our study to be justified [16,27]. However, an age-matched control would allow investigation of the extent of recovery, with the interventions investigated, when compared to uninjured normal bones. Including an age-matched control group proved to be challenging in this study as the treadmill training alone, transplant alone and combinational group animals had different euthanasia timelines and were sacrificed at Week 9, Week 10 and Week 11, respectively. An age-matched sham animal group would require 24 additional animals to have a comparative baseline for each group. Based on these findings, we decided to compare all treatment groups to the injury group with the goal of reporting any recovery/improvement and not restoration to a control/sham animal. We also recommend future studies to include a larger sample size to better characterize the efficacy of combinational therapy. For the studied bone micro-structural parameters, the reported *p* values were >0.685 in the current study. For studied bone biomechanical parameters, the reported *p* values were >0.716 in the current study. Future studies should carefully account for the sample size and statistical analysis required to confirm the effects of the combinatorial treatment strategy. Overall, despite the lack of statistical significance, this study is the first to report the effects of combinational treatment strategies and serves as a guide for future studies that can further investigate the efficacy of the combinational treatment strategy on bone loss in SCI animal models across various species. Studies should also investigate the timing of interventions (acute versus delayed) and explore the critical windows for intervention post-SCI. Utilizing additional behavioral tests, physiological assessment and biochemical analysis that help evaluate motor function, pain and other functional outcome while elucidating the underlying mechanism of recovery will further support a combinational treatment strategy as a promising treatment modality for curing SCI.

## Figures and Tables

**Table 1:** Animal groups and sample size details.

Groups (n = 8 Each)	Description
Injury (Gr 1)	Injured (SCI + No Treatment)
BWSTT (Gr 2)	BWSTT (SCI + BWSTT)
Scaffold (Gr 3)	PNIPAAM-g-PEG+BDNF+NT3 (SCI + Scaffold releasing Neurotrophins)
Combinational (Gr 4)	Combinational (SCI + Scaffold releasing Neurotrophins + BWSTT)

**Table 2:** Study timeline including the injury, treatment type and harvest time-point details for each group.

Groups (n = 8)	Week 0	Week 1	Week 2	Week 8	Week 9	Week 10
Injury (Gr 1)	T9/10 Contusion SCI			Harvest		
BWSTT (Gr 2)		BWSTT			Harvest	
Scaffold (Gr 3)		Transplant surgery			Harvest	
Combinational (Gr 4)		Transplant surgery	BWSTT			Harvest

## Data Availability

Data is unavailable due to ethical restrictions but can be made available upon request and approval from the institution.
